# Hyponatremia-related liver steatofibrosis and impaired spermatogenesis: evidence from a mouse model of the syndrome of inappropriate antidiuresis

**DOI:** 10.1007/s40618-022-01962-9

**Published:** 2022-11-27

**Authors:** G. Marroncini, C. Anceschi, L. Naldi, B. Fibbi, M. Brogi, L. Lanzilao, A. Fanelli, M. Maggi, A. Peri

**Affiliations:** 1 Pituitary Diseases and Sodium Alterations Unit, AOU Careggi, 50139 Florence, Italy; 2 Department of Experimental and Clinical Biomedical Sciences “Mario Serio”, University of Florence, AOU Careggi, Viale Pieraccini, 6, 50139 Florence, Italy; 3 Endocrinology, Department of Geriatric Medicine, Careggi University Hospital, 50139 Florence, Italy; 4 Central Laboratory, Careggi University Hospital, 50139 Florence, Italy

**Keywords:** Hyponatremia, SIAD, Liver steatosis, Spermatogenesis, Oxidative stress

## Abstract

**Purpose:**

Hyponatremia is the most frequent electrolytic disorder in clinical practice. In addition to neurological symptoms, hyponatremia, even when mild/moderate and chronic, has been related to other manifestations, such as bone demineralization and increased risk of fractures. To better elucidate tissue alterations associated with reduced serum sodium concentration [Na^+^], we developed an in vivo model of hyponatremia secondary to the Syndrome of Inappropriate Antidiuresis.

**Methods and results:**

Hyponatremia was induced in Foxn1^nu/nu^ mice by subcutaneous infusion of the vasopressin analog 1-deamino [8-D-arginine] vasopressin (dDAVP) for 14 days via osmotic mini-pumps. Mice in the control group were infused with isotonic saline solution. Serum [Na^+^] progressively decreased, with a nadir of 123.4 ± 2.3 mEq/L (mean ± SD, dDAVP 0.3 ng/h) and 111.6 ± 4.7 mEq/L (mean ± SD, dDAVP 0.5 ng/h). Evident signs of liver steatofibrosis were observed at histology in hyponatremic mice. Accordingly, the expression of proteins involved in lipid metabolism (SREBP-1, PPARα and PPARγ) and in myofibroblast formation (αSMA and CTGF) significantly increased. Furthermore, heme oxygenase 1 expression was up-regulated in Kupffer and hepatic stellate cells in the liver of hyponatremic mice. Testis alterations were also observed. In particular, the thickness of the seminiferous epithelium appeared reduced. The expression levels of PCNA and PTMA, which are involved in DNA replication and germ cells maturation, were markedly reduced in the testis of hyponatremic mice.

**Conclusion:**

Overall, these findings shed new light on the possible consequences of chronic hyponatremia and prompt a more thorough evaluation of hyponatremic patients.

## Introduction

Hyponatremia, defined by a serum sodium concentration [Na^+^] of less than 135 mEq/L, is the most frequent electrolyte alteration in clinical practice [1] and it is more common in the elderly, with incidences that vary from 7 to 53% in this subgroup [2–5]. In more than 40% of cases, hyponatremia is due to the Syndrome of Inappropriate AntiDiuresis (SIAD) [1, 6]. Hyponatremia, especially when acute (i.e., onset from less that 48 h) and severe (serum [Na^+^] < 120 mEq/L), has been associated with evident neurological symptoms, which are secondary to brain edema [7, 8]. However, also chronic and mild/moderate hyponatremia (serum [Na^+^] 126–135 mEq/L), once considered asymptomatic, have been more recently re-evaluated and associated with a variety of symptoms. They include, for instance, cognitive disorders, gait instability and increased risk of falls [9, 10]. Furthermore, mild hyponatremia has been associated with an increased length of stay in the hospital, risk of hospital readmission and death in several clinical conditions, including cancer [11–15].

Animal models can further clarify the consequences of low serum [Na^+^]. In 1984, a rat model of chronic hyponatremia secondary to SIAD was developed by the group of Joseph Verbalis [16]. In these animals, a condition mimicking SIAD was obtained by continuous injection of 1-desamino-8-D-arginine vasopressin (dDAVP) together with a liquid diet. Interestingly, more recent evidence revealed that hyponatremic rats develop bone demineralization, with approximately a 30% mineral density reduction after 3 months, compared with normonatremic rats [17]. These data were confirmed in humans. In particular, the analysis of data from the Third National Health and Nutrition Examination Survey (NHANES III) indicated that hyponatremia is associated with an increased risk of osteoporosis at the hip [17]. Subsequent in vitro studies showed that low extracellular [Na^+^] determines osteoclast activation on one hand [18], and inhibition of osteogenesis on the other hand [19]. Further analysis of male hyponatremic rats showed that these animals developed primary hypogonadism, abnormal testicular histology, sarcopenia and cardiomyopathy [20]. The authors concluded that hyponatremia elicits multiple manifestations of senescence in male rats.

A mouse model of hyponatremia secondary to SIAD has been rarely reported, so far. In two studies, dDAVP infusion in mice was used to induce hyponatremia. However, because the aim of these studies was to investigate on osmotic demyelination, hyponatremia was rapidly corrected by hypertonic saline infusion 4 days later [21, 22]. Only very recently, a mouse model of sustained hyponatremia due to SIAD was developed. In these mice, behavioral analyses were performed and confirmed the presence of cognitive impairment, particularly related to loss of memory [23].

However, to date, several areas of possible tissue alterations associated with chronic hyponatremia remain unexplored. For this reason, we have developed a mouse model of chronic hyponatremia secondary to SIAD. In our experimental design, we used Foxn1^nu/nu^ mice, because this mouse model is intended to be used in upcoming projects, to study tumor proliferation and spread in normonatremia vs. hyponatremia. Here, we present data on tissue alterations observed in the liver and testis of hyponatremic mice.

## Materials and methods

### Animal model of hyponatremia

All animal experiments were conducted according to institutional ethical norms and national laws after approval from the Italian Ministry of Health [D. N° 688/2020-PR (prot. 17E9C.201)] [24]. 8-week-old Foxn1^nu/nu^ male mice (Charles River Laboratories International, Wilmington, Massachusetts, USA) were housed in a standard animal facility (Ce.S.A.L., Department of Biomedical, Experimental and Clinical Sciences “Mario Serio”, Florence, Italy) in sterile areas with a 12/12 h light/dark cycle and a constant temperature (21–23 °C), equipped with ventilation and sterile barriers inside “sterile filter top” cages. In the first week of acclimatization, the animals (25–30 g of body weight) had ad libitum access to standard chow (MF^®^; Oriental Yeast Co., Ltd., Tokyo, Japan) and tap water. Then mice were randomly divided into three experimental groups: a control group (*n* = 15) and two treatment groups, group A and group B (*n* = 15 each one).Subsequently, according to the previously described hyponatremic rat model [16], mice were fed with a nutritionally balanced (66% carbohydrates, 21% protein, 12% fat and vitamins) rodent liquid formula (Rodent Liquid Diet AIN-76A, Mucedola S.R.L., Milan, Italy) and tap water for seven days. After this time, osmotic mini-pump (model 1002, Alzet, Cupertino, CA, USA) was implanted. Mice in the control group were subcutaneously infused with isotonic saline (0.9% NaCl), whereas mice that received dDAVP (MINIRIN/DDAVP 0.1 mg/ml, Ferring S.P.A., Milan, Italy) in isotonic saline solution were infused at two different rates: 0.3 ng/h (group A) and 0.5 ng/h (group B). To decrease the amount of fluid ingested, from the day of implantation and for the entire duration of the experiment (14 days), treatment groups were fed only with rodent liquid diet without access to tap water.

### Analysis of serum and urine

The experimental design included sacrifice of control and dDAVP-treated mice (n = 3 for each group) at different time points, to assess the induction and maintenance of hyponatremia by serum and urine analysis. In 5 animals, blood was drawn by venipuncture (submandibular vein) and urine was collected at time T-3. Considering as time point zero (T0), the day of the implantation of the osmotic mini-pumps, at time points T0, T3, T8, T14 and T14 + 3, mice were sacrificed with an overdose of anesthetic (ketamine/xylazine) necessary to take blood via cardiocentesis-transthoracic. The day before the sacrifice, the animals were housed individually in metabolic cages and urines were collected and analyzed for osmolality (Model 3320 Osmometer, Advanced Instrument Inc., New Taipei City, Taiwan) and [Na^+^]. After death, blood samples were collected via cardiocentesis-transthoracic, centrifuged at 1500 rpm for 20 min at + 4 °C and then processed for osmolality (Model 3320 Osmometer, Advanced Instrument Inc., New Taipei City, Taiwan) and [Na^+^] measurement using the Cobas 8000 (Roche/Hitachi family, Basel, Switzerland). The biochemical analyses were carried out by the General Clinical Chemical laboratory of AOU Careggi, Florence, according to the standard procedures.

### Testosterone, luteinizing hormone and follicle-stimulating hormone analysis

Testosterone, luteinizing hormone (LH) and follicle-stimulating hormone (FSH) concentrations were determined. In particular, testosterone concentrations were measured using the Cobas 8000 (Roche/Hitachi family, Basel, Switzerland) at the General Clinical Chemical laboratory of AOU Careggi, whereas FSH and LH were measured by two competitive ELISA kits: mouse follicle-stimulating hormone ELISA kit (RRID: AB_2920724, CSB-E06871m, Cusabio Technology LLC, Houston, TX, USA) and mouse luteinizing hormone ELISA kit (RRID: AB_2920725, CSB-E12770m, Cusabio Technology LLC, Houston, TX, USA), according to manufacturers’ instructions. Briefly, after blood samples’ centrifugation, 50 µl of serum was added to 50 µl of specific secondary antibody in 96-well ELISA plates and incubated at 37 °C for one hour. Then, 50–100 µl of HRP conjugate substrates were put into the well and incubated before adding stop solution. The optical density was measured through a microplate reader set to 450 nm.

### Tissue preparation and morphological characterization

After blood drawing, tissues were rapidly explanted, partly fixed in 10% formalin (65-30001F—Bio-Optica Milano Spa, Milan, Italy) for at least 48 h and partly cryopreserved. Formalin fixed tissues were washed in water for at least 24 h before being automatically embedded in paraffin through the ASP300S and HistoCore processor (Arcadia Inclusion System, Leica Biosystems, Milan, Italy). Tissues were then sectioned in 5–7 µm slices and put on Superfrost glasses (630-2835, Thermo Fisher Scientific, Waltham, Massachusetts, USA). In the present study, liver and testis tissues were analyzed. After de-paraffinization in xylene and dehydration in decreasing series of alcohols, 5–7 µm of liver and testis sections was stained with haematoxylin (Hematoxylin Gill 3, 05-06015L—Bio-Optica Milano Spa, Italy) and eosin (Eosin Y alcoholic solution, 05-10,003/L—Bio-Optica Milano Spa, Italy). At the same time, liver sections were also stained with Sirius Red (Sirius red F3B—Sigma–Aldrich, St. Louis, Missouri, USA) to highlight collagen fibers. Finally, all slides were dehydrated and mounted in a resinous medium (09-00500, Eukitt—BioOptica Milano Spa, Italy). As for frozen tissues, 5–7 µm sections from liver were stained with Oil Red-O to assess lipid content. Moreover, 10 µm sections from liver and testis were homogenized in 500 µl of RIPA lysis buffer (50 mM Tris–HCl pH 7.5, 120 mM NaCl, 1 mM EDTA, 6 mM EGTA, 15 mM Na_4_P_2_O_7_, 20 mM NaF, 1% NP-40) supplemented with protease (11,697,498,001, Roche, Basel, Switzerland) and phosphatase inhibitor cocktail (#5870, Cell Signaling Technology, Danvers, Massachusetts, USA) 100X, to obtain whole protein lysate for immunoblot analysis.

### Western blot analysis

Equal amount of liver and testis proteins (10–15 ug) was separated on TGX Stain-Free FastCast Acrylamide Kit 10% (#1,610,183, Bio-Rad) by electrophoretic run. The samples were transferred onto PVFD membrane (Immobilion, Billerica, Millipore, MA, USA) and were subsequently blocked with milk 5% for 1 h. Afterward, the membranes were incubated overnight at + 4 °C with primary antibodies: rabbit polyclonal anti-HMOX1 (RRID:AB_880536, SAB2108676, 1:1000, Sigma–Aldrich, St. Louis, Missouri, USA), mouse monoclonal anti-PCNA (RRID:AB_2160343, #2586S, 1:2000, Cell Signaling Technology, Danvers, Massachusetts, USA), mouse monoclonal anti-PPARγ (RRID: AB_2920698, sc-390740, 1:1000, Santa Cruz Biotechnology, Dallas, Texas, USA), mouse monoclonal anti-PPARα (RRID:AB_2885073, sc-398394, 1:1000, Santa Cruz Biotechnology, Dallas, Texas, USA), mouse monoclonal anti-CTGF (RRID:AB_10917205, sc-373936, 1:1000, Santa Cruz Biotechnology, Dallas, Texas, USA), mouse monoclonal anti-α-SMA (RRID:AB_476701, A2547, 1:1000, Sigma–Aldrich, St. Louis, Missouri, USA) and mouse monoclonal anti-SREBP1 (RRID:AB_10843812, sc-365513, 1:1000, Santa Cruz Biotechnology, Dallas, Texas, USA). The day after, membranes were washed twice in PBST and incubated with the specific secondary antibody (HRP-linked anti-mouse IgG, RRID:AB_330924, #7076 Cell Signaling Technology, Danvers, Massachusetts, USA or HRP-linked anti-rabbit IgG, RRID:AB_2536530, G-21234 Invitrogen, Waltham, Massachusetts, USA) conjugated to horseradish peroxidase for 1 h at room temperature. After peroxidase substrate incubation (Immobilon Crescendo Western HRP substrate, WBLUR0500, Millipore, Burlington, Massachusetts, USA), chemiluminescent images were acquired with a BioRad ChemiDoc Imaging System (Biorad, Hercules, CA, USA) and analyzed with ImageJ Software Gel. Images were normalized versus the whole quantity of protein loaded in the gel visualized with stain free system.

### Immunohistochemical analysis

After de-paraffinization and rehydration, formalin-fixed slices were boiled in Buffer Citrate (pH = 6) at 95 °C for 10 min for antigenic unmasking of samples. To inhibit tissue peroxidases, slices were placed in 6% H_2_O_2_ solution for 30 min at room temperature and then blocked in PBS–BSA (bovine serum albumin) 2% solution for 1 h. To reduce the binding of endogenous antibodies, slices then were incubated with ReadyProbes™ Mouse-on-Mouse IgG Blocking Solution (R37621, Invitrogen, Waltham, Massachusetts, USA) for 1 h at room temperature. Subsequently, the slices were incubated overnight at 4 °C with the primary antibody rabbit polyclonal anti-HMOX1 (RRID: AB_880536, ab52947, 1:100, Abcam, Cambridge, UK), rabbit polyclonal anti-F480 (RRID:AB_2881149, 28,463-1-AP, 1:2000, Proteintech Europe, Manchester, UK), mouse monoclonal anti-α-SMA (RRID:AB_2223500, M0851, 1:100, Dako Agilent Technologies, Santa Clara, California, USA), mouse monoclonal anti-PCNA (#2586, 1:16,000, Cell Signaling Technology, Danvers, Massachusetts, USA), rabbit polyclonal anti-PTMA (RRID:AB_2679972, HPA047183, 1:50, Sigma–Aldrich, St. Louis, Missouri, USA). Anti-F480 and anti-α-SMA were kindly provided by Drs. Tommaso Mello and Simone Polvani (Gastroenterology Unit, Department of Experimental and Clinical Biomedical Sciences “Mario Serio”, University of Florence, Italy). Finally, slices were incubated for 1 h with specific secondary antibody conjugated to horseradish peroxidase (HRP-linked anti-mouse IgG, #7076 or HRP-linked anti-rabbit IgG, RRID:AB_2099233, #7074 Cell Signaling Technology, Danvers, Massachusetts, USA). AEC (3-amino-9-ethylcarbazole) Substrate Peroxidase (HRP) Kit (RRID:AB_2336076, SK-4200, Vector Laboratories, Burlingame, CA, USA) and SignalStain^®^ DAB Substrate Kit (#8059, Cell Signaling Technology, Danvers, Massachusetts, USA) were used for antigen detection. In addition, slices were counter-stained with Gill’s haematoxylin, to visualized cells nuclei. The slices were mounted in resinous medium. For each one, five fields of view were randomly selected under an optical microscope and AEC/DAB positive cells were analyzed and quantified using imageJ software and GraphPad Prism 5.0 Software.

### Statistical analysis

Each experiment was performed in triplicates unless otherwise stated. Statistical analysis was performed with GraphPad. Normality of data distribution was assessed with the Shapiro–Wilk normality test and differences in experimental results were evaluated by Student's *t* test or the Mann–Whitney *U* test (Wilcoxon rank-sum test), for parametric and non-parametric data, respectively. When comparing multiple groups, ANOVA followed by Dunn, highly significant differences post hoc test was used for parametric data, whereas the Kruskal–Wallis test followed by the Conover-Iman test was used for pairwise comparisons of non-parametric data. Values are expressed as the mean ± standard deviation (SD), and *p* ≤ 0.05 was considered to indicate statistical significance.

## Results

### Induction of hyponatremia in mice

**Fig. 1:**
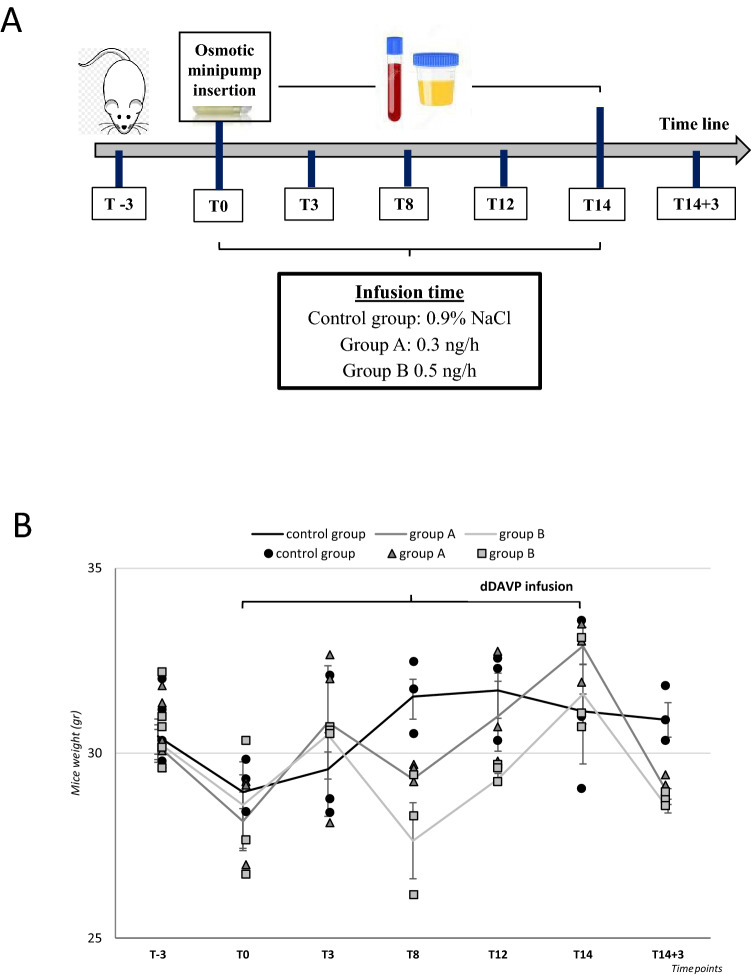
Experimental protocol and weight fluctuations. **a** Protocol used for the mouse model of hyponatremia. All the animals were fed with a rodent liquid diet. Animals were infused with two different rates of dDAVP (group A and B) for 14 days (T0-T14) via osmotic mini-pumps, whereas the control group with 0.9% NaCl. Animals were sacrificed at different time points and blood and urine samples were obtained. **b** Weight fluctuations of the three experimental groups. Results are expressed as mean ± SD

Hyponatremia was induced by administering a liquid diet and subcutaneous infusion of dDAVP for 14 days (T0–T14), as described in detail in Materials and Methods. Two different rates of infusion of dDAVP were used (0.3 ng/h, *n* = 15, group A; and 0.5 ng/h, *n* = 15, group B). One group of mice (*n* = 15), which was fed with the same diet and was infused with isotonic saline solution, was used as the control. The experimental protocol is represented in Fig. 1a.

Weight fluctuations were observed during the experimental procedure. A trend toward a weight reduction was observed in all groups of mice after the initiation of a liquid diet (from T-3 to T0). dDAVP administration was associated with a significant increase of weight in both group A (28.2 ± 0.8 g T0, vs. 32.9 ± 0.5 g, T14, *p* ≤ 0.05) and B (28.6 ± 1.2 g, T0, vs. 31.6 ± 0.8 g, T14, *p* ≤ 0.05). Conversely, no significant weight difference was observed in the control group at the end of isotonic saline (0.9% NaCl) infusion (28.95 ± 0.4 g, T0, vs. 31.1 ± 1.4 g, T14) (Fig. 1b, Table 1). After the discontinuation of dDAVP administration, a significant weight loss was observed (T14 + 3) and the final weight of animals in both group A and B was virtually identical to the initial weight (T0).

**Table 1:** Weight fluctuations

	Mice weight (gr)		
Days	Control group	Group A	Group B
T-3	30.5 ± 0.5	30.5 ± 0.5	30.5 ± 0.5
T0	28.95 ± 0.4	28.2 ± 0.8	28.6 ± 1.2
T3	29.6 ± 1.3	30.8 ± 1.5	30.5 ± 0.1
T8	31.5 ± 0.6*	29.3 ± 0.2	27.6 ± 1
T12	31.7 ± 0.8*	31 ± 0.9*	29.3 ± 0.2
T14	31.1 ± 1.4	32.9 ± 0.5*	31.6 ± 0.8*
T14 + 3	30.9 ± 0.5^#^	28.9 ± 0.1^#^	28.5 ± 0.1^#^

*p ≤ 0.05 vs. T0; ^#^*p* ≤ 0.05 vs. T14

**Fig. 2:**
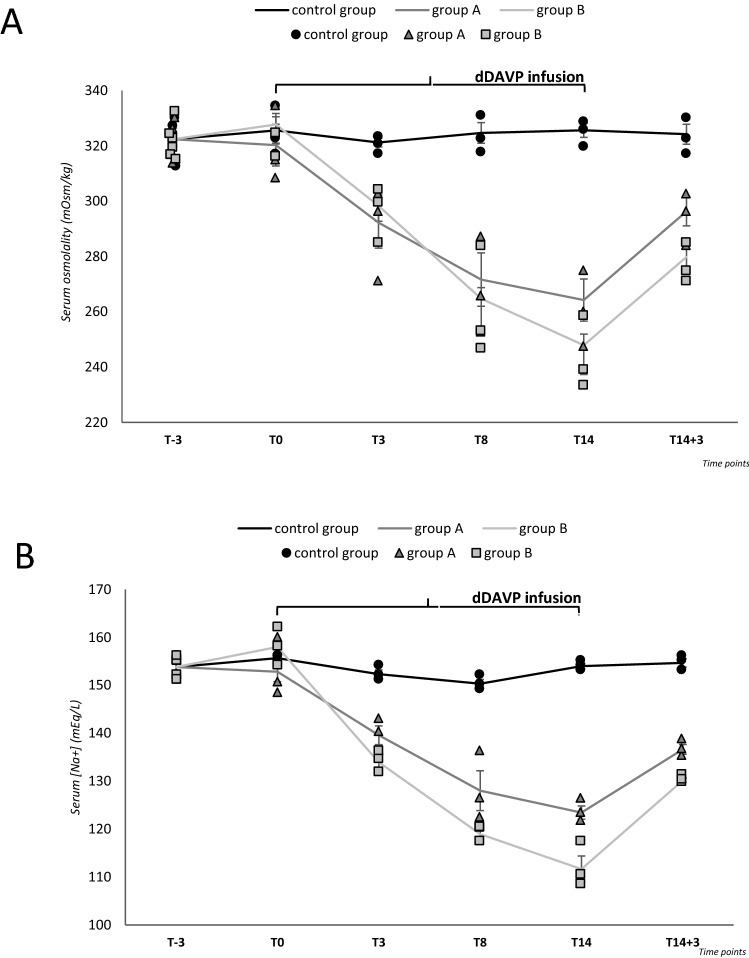
Analysis of serum osmolality and serum [Na^+^]. Blood was collected by transthoracic cardiocentesis at the time of sacrifice. **a** Serum osmolality (mOsm/kg) in group A and B and in the control group. **b** Serum [Na^+^] (mEq/L) in group A and B and in the control group. Results are expressed as mean ± SD

A progressive reduction of serum [Na^+^] was observed in animals receiving dDAVP (152.8 ± 2.1 mEq/L, group A and 158 ± 1.4 mEq/L, group B, at T0), with a serum [Na^+^] nadir of 123.4 ± 1.4 mEq/L and 111.7 ± 2.7 mEq/L in group A and B, respectively, at T14, *p* ≤ 0.05 vs. T0 (Fig. 2a, Table 2). In the 3 days following dDAVP discontinuation, a serum [Na^+^] increase was observed, as expected (136.6 ± 1 mEq/L and 130.2 ± 0.4 mEq/L in group A and B, respectively, at T14 + 3 vs. T14). Conversely, in control animals, no difference in serum [Na^+^] was observed between T0 and T14 (155.7 ± 0.2 mEq/L vs. 154 ± 0.6 mEq/L).

**Table 2:** Serum osmolality and [Na^+^]

	Serum [Na^+^] (mEq/L)			Serum osmolality (mOsm/kg)		
Days	Control group	Group A	Group B	Control group	Group A	Group B
T-3	153.8 ± 1.2	153.8 ± 1.2	153.8 ± 1.2	322.4 ± 2.1	322.4 ± 2.1	322.4 ± 2.1
T0	155.7 ± 0.2	152.8 ± 2.1	158 ± 1.4	325.6 ± 4.9	320.3 ± 7.5	327.8 ± 6.7
T3	152.3 ± 0.9	139.6 ± 2^#^	134 ± 1.3*^#^	321.2 ± 1.6	292.3 ± 9.2^#^	298.3 ± 5.6^#^
T8	150.3 ± 0.9^#^	128 ± 4.2^#^	118.9 ± 1*^#^	324.7 ± 3.7	271.7 ± 9.6*^#^	264.8 ± 11^#^
T14	154 ± 0.6	123.4 ± 1.4^#^	111.7 ± 2.7*^#^	325.6 ± 2.5	264.2 ± 7.6^#^	251.3 ± 10.6^#^
T14 + 3	154.7 ± 0.9	136.6 ± 1	130.2 ± 0.4	324.2 ± 3.6	296.3 ± 5.3	279.8 ± 4

*p ≤ 0.05 vs. control group; ^#^*p* ≤ 0.05 vs. T0

With regard to serum osmolality, a significant decrease was observed in group A and B after starting dDAVP infusion, with a nadir at T14 (264.2 ± 7.6 mOsm/kg, group A, and 251.3 ± 10.6 mOsm/kg, group B, *p* ≤ 0.05 vs. T0) (Fig. 2b, Table 2). No variation was observed in control animals.

**Fig. 3:**
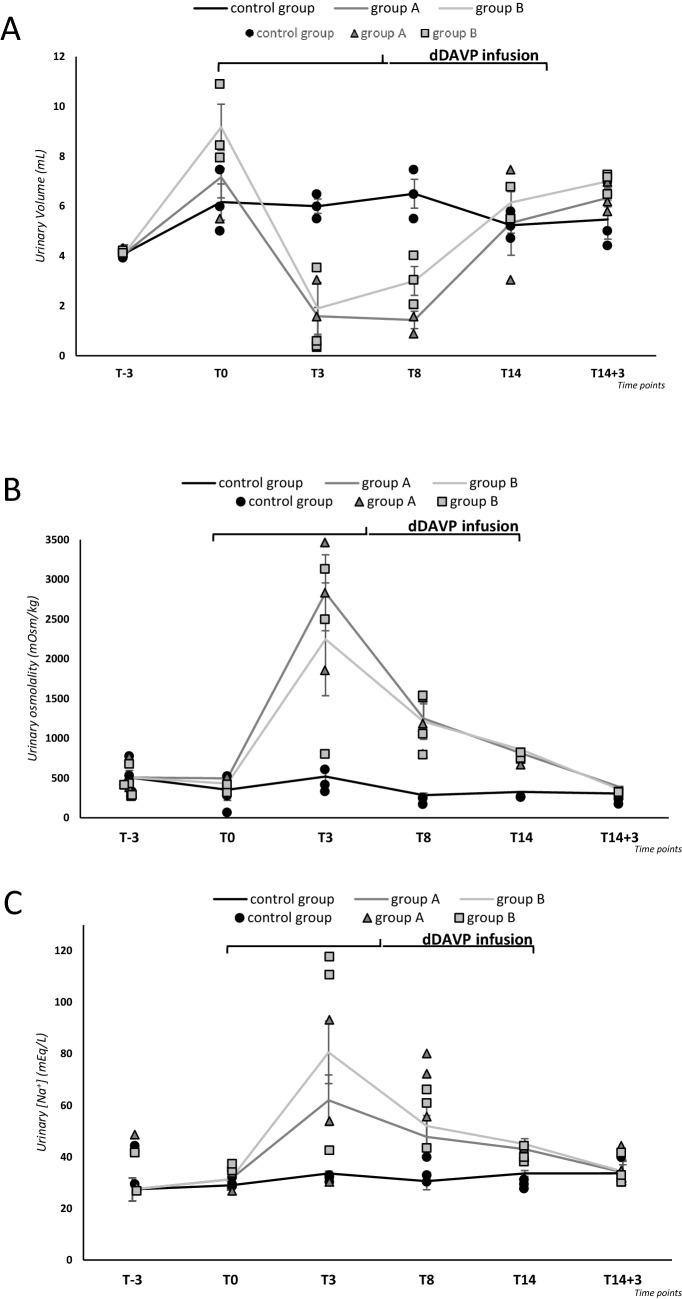
Analysis of urinary volume, urinary osmolality and urinary [Na^+^]. Urinary volume (mL) (**a**), osmolality (mOsm/kg) (**b**) and [Na^+^] (mEq/L) (**c**) in group A and B and in the control group, measured by housing animals in metabolic cages for 24 h. Results are expressed as mean ± SD

Urinary volume increased, yet not significantly, upon starting administration of a liquid diet in all groups but was restrained once dDAVP infusion was initiated (group A and B), with a nadir at T3. A urinary volume increase was again observed at the end of dDAVP administration. (Fig. 3a, Table 3). Together with the marked reduction of urinary volume following the initiation of dDAVP infusion, a significant increase of urine osmolality was observed, with a peak at T3 (Fig. 3b, Table 3). In agreement with urinary volume changes, urinary osmolality decreased at the end of dDAVP administration. Conversely, no significant change of urinary volume and osmolality was observed in the control group.

**Table 3:** Urinary volume, osmolality and [Na^+^]

	Urinary volume (mL)			Urinary osmolality (mOsm/kg)			Urinary [Na^+^] (mEq/L)		
Days	Control group	Group A	Group B	Control group	Group A	Group B	Control group	Group A	Group B
T-3	4.1 ± 1	4.1 ± 1	4.1 ± 1	508.8 ± 88.5	508.8 ± 88.5	508.8 ± 88.5	27.4 ± 4.5	27.4 ± 4.5	27.4 ± 4.5
T0	6.2 ± 0.7	7.2 ± 0.8	9.1 ± 0.9	353.3 ± 134.8	497.3 ± 46.9	431.7 ± 29.7	29 ± 1.5	31.3 ± 3.3	31.3 ± 4.3
T3	6 ± 0.3	0.7 ± 0.4^#^	1.9 ± 1^#^	519.7 ± 83.5	2833 ± 478.5^#^	2247.3 ± 710.6^#^	30.7 ± 0.9	62 ± 9.8	80.6 ± 12.1
T8	6.5 ± 0.6	1.4 ± 0.3*^#^	3 ± 0.6^#^	285.3 ± 26.8	1250.3 ± 209.7^#^	1211.7 ± 222.4^#^	33.7 ± 3.3	47.8 ± 8.3	52.3 ± 7.9
T14	5.2 ± 0.3	5.3 ± 1.3	6.1 ± 0.4	326.3 ± 2.4	811.3 ± 40.8	857 ± 21.4	28 ± 1.2	43 ± 1.5*	45 ± 2.1
T14 + 3	5.5 ± 0.8	6.3 ± 0.4	7 ± 0.3	306 ± 43.5	359 ± 9.1	388 ± 2.1	33.7 ± 3.3	34 ± 4.7	34.3 ± 3.9

*p ≤ 0.05 vs. control group; ^#^*p* ≤ 0.05 vs. T0

Finally, there was a trend to an increase of urinary [Na^+^] in mice from group A and B, with a peak at T3 and a progressive decrease afterward. No variation in urinary [Na^+^] was observed in the control group (Fig. 3c, Table 3).

### Liver alterations

**Fig. 4:**
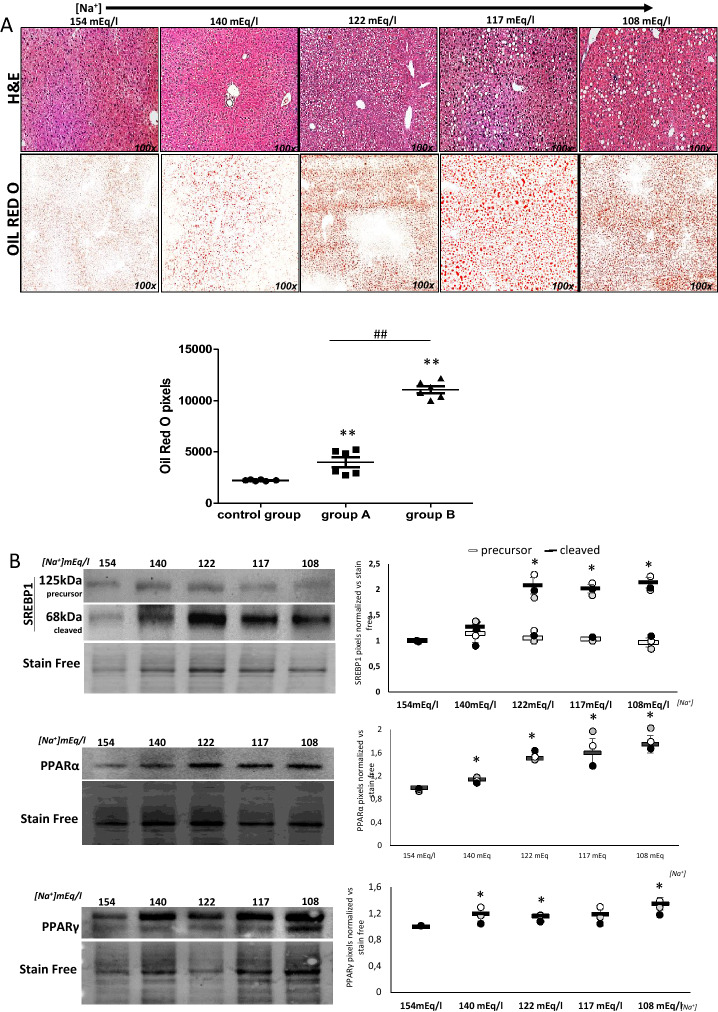

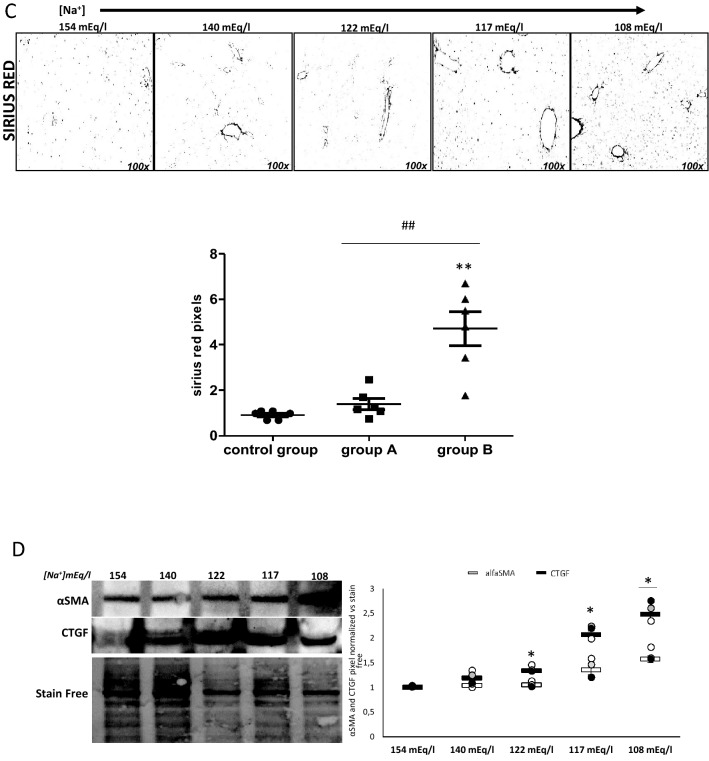
Analysis of liver from control mice and hyponatremic mice. **A** Representative images of liver sections. Steatosis is highlighted by hematoxylin–eosin and Oil red O staining. In the scatter plot, densitometric analysis of positive pixels of lipid droplets is represented (**p ≤ 0.02 group A and B vs. control group; ^##^*p* ≤ 0.02, group A vs. B). Results are expressed as mean ± SD. **B** SREBP-1, PPAR-α and PPAR-γ expression were analyzed by Western blot. Representative experiments are shown on the left, the results of three different experiments for each protein on the right. Results are expressed as mean ± SD. (*p ≤ 0.05 vs. 154 mEq/L). **C** Liver fibrosis is highlighted by Sirius red staining. In the scatter plot, densitometric analysis of positive pixels of the fibrotic areas is represented (**p ≤ 0.02 group A and B vs. control group; ^##^*p* ≤ 0.02, group A vs. B). Results are expressed as mean ± SD. **D** Western Blot analysis of fibrosis markers in liver: α-SMA and CTGF proteins. Representative experiments are shown on the left, the results of three different experiments for each protein on the right. Results are expressed as mean ± SD. (*p ≤ 0.05 vs. 154 mEq/L). For immunohistochemical images and Western blot analysis, [Na^+^] 154 mEq/L is from control group; [Na^+^] 140 and 122 mEq/L are from group A, T3 and T8, respectively; [Na^+^] 117 and 108 mEq/L are from group B, T8 and T14, respectively

For the analysis of tissue alterations, mice sacrificed at different time points (T0, T3, T8, T14) from group A, group B, and control group were used. Evident signs of steatosis were observed in the liver of hyponatremic mice, as indicated by the presence of ballooning degeneration of hepatocytes in hematoxylin–eosin stained tissue samples and by the increased number of lipid droplets labeled by Oil Red-O staining. Noticeably, liver steatosis progressively increased as serum [Na^+^] dropped down, together with a progressive accumulation of lipid droplets, which was significantly different in group A and B vs. control group and in group A vs. group B (*p* ≤ 0.02). (Fig. 4a). Morphological changes were substantiated by the increased expression of the cleaved (i.e., mature) form of the Sterol Regulatory Element-Binding Protein 1 (SREBP-1), which plays a crucial role in the induction of liver lipogenesis (Fig. 4b). Similarly, the expression of the Peroxisome Proliferator-Activated Receptor alpha (PPAR-α) and, yet to a lesser extent, gamma (PPAR-γ), two major regulators of lipid metabolism in the liver, was increased in hyponatremic mice (Fig. 4b).

Together with the presence of steatosis, in mice with severe hyponatremia (group B), liver fibrosis was observed, as indicated by Sirius Red staining (*p* ≤ 0.02 vs. control group) (Fig. 4c). Accordingly, the expression of alpha-Smooth Muscle Actin (α-SMA) and of Connective Tissue Growth Factor (CTGF), which are markers of myo-fibroblast formation, increased (Fig. 4d).

**Fig. 5:**
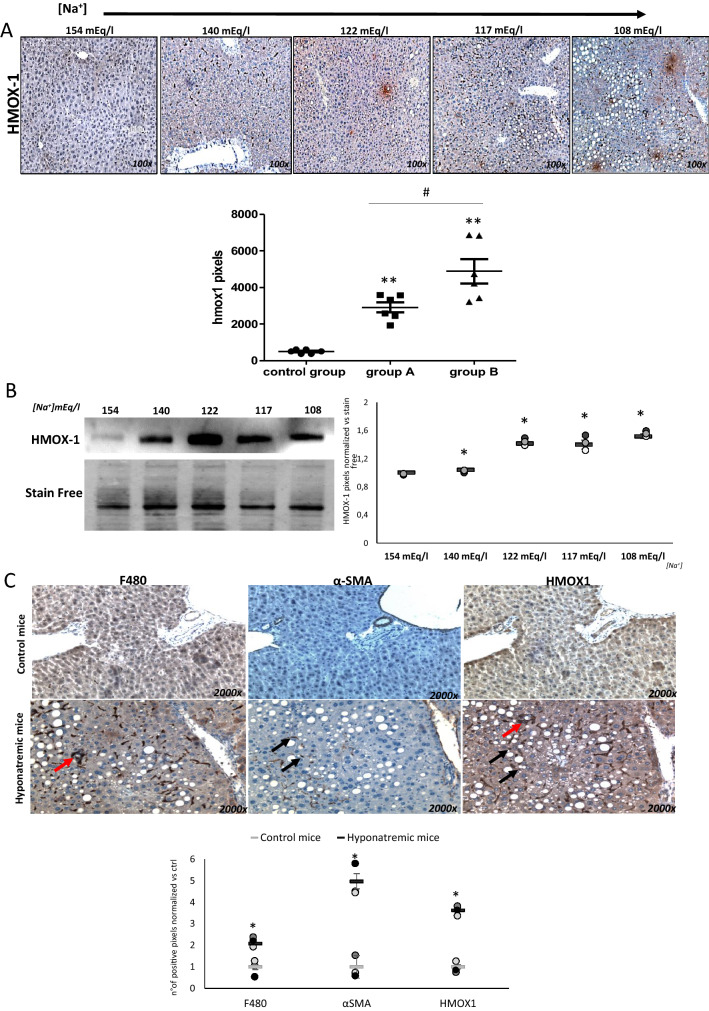
Expression analysis of HMOX-1 in liver of control mice and hyponatremic mice. **A** Immunohistochemical analysis of HMOX-1. In the scatter plot, densitometric analysis of positive pixels of HMOX-1 positive cells is represented (**p ≤ 0.02 group A and B vs. control group; ^#^*p* ≤ 0.05, group A vs. B). Results are expressed as mean ± SD. **B** Western Blot analysis of HMOX-1 expression in liver. A representative experiment is shown on the left, the results of three different experiments on the right. Results are expressed as mean ± SD (*p ≤ 0.05 vs. 154 mEq/L). **C** Images related to serial immunohistochemical staining of F480, α-SMA and HMOX-1, indicating that HMOX-1 protein is expressed by both Kupffer cells (red arrows) and HSCs (black arrows). In the scatter plot, densitometric analysis of positive pixels in mice with [Na^+^] ≤ 140 mEq/L (hyponatremic mice) normalized vs. mice from control group is represented. Results are expressed as mean ± SD (*p ≤ 0.05). For immunohistochemical images and Western blot analysis, [Na^+^] 154 mEq/L is from control group; [Na^+^] 140 and 122 mEq/L are from group A, T3 and T8, respectively; [Na^+^] 117 and 108 mEq/L are from group B, T8 and T14, respectively

Finally, Heme Oxygenase 1 (HMOX 1) gene expression, which was virtually nil in the liver of normonatremic mice, significantly increased in hyponatremic animals, as shown by immunohistochemistry (*p* ≤ 0.02 vs. control group and *p* ≤ 0.05 between group A and B) and Western blot analysis (Fig. 5a, b). To identify the nature of cells expressing HMOX 1 in the liver of hyponatremic animals, serial liver sections were subjected to immunohistochemistry analysis for F480, a marker of activated Kupffer cells in the liver [25, 26], and αSMA, a marker of myo-fibroblast formation, as previously mentioned, which is expressed by activated Hepatic Stellate Cells (HSC) [27]. Very low (F480) or no immuno-positivity (α-SMA) were found in the liver of normonatremic mice, whereas evident positive staining was observed for both markers in liver sections of hyponatrenic mice (Fig. 5c). The comparison between serial sections suggested that the cells expressing HMOX 1 in low [Na^+^] correspond to activated Kupffer cells and HSC.

### Testis alterations

**Fig. 6:**
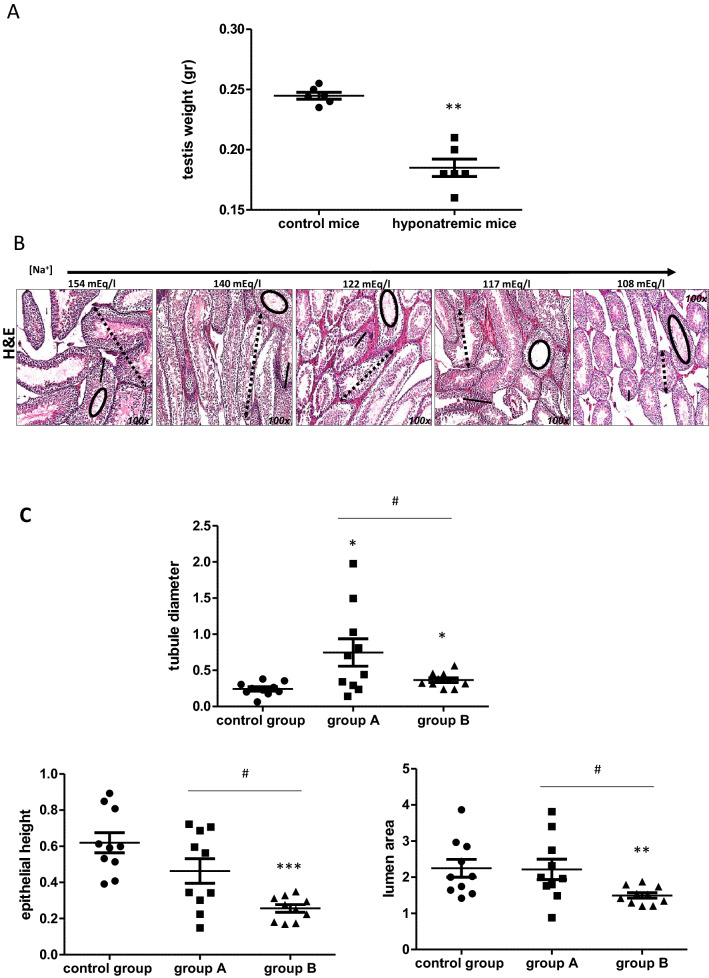
Weight and histology of testes from control mice and hyponatremic mice. **A** Testis weight (gr) of mice with [Na^+^] ≤ 140 mEq/L (hyponatremic mice) normalized vs. mice from control group. Results are expressed as mean ± SD (**p ≤ 0.02). **B** Hematoxylin–eosin staining of testis sections. Tubule diameter is represented by dotted lines, lumen area by circles and epithelial height by continuous lines. [Na^+^] 154 mEq/L is from control group; [Na^+^] 140 and 122 mEq/L are from group A, T3 and T8, respectively; [Na^+^] 117 and 108 mEq/L are from group B, T8 and T14, respectively. **C** Measurements are represented by scatter plots (*p ≤ 0.05 group A and B vs. control group; **p ≤ 0.02 group A and B vs control group; ***p ≤ 0.002 group A and B vs. control group; ^#^*p* ≤ 0.05, group A vs. B). Results are expressed as mean ± SD

**Fig. 7:**
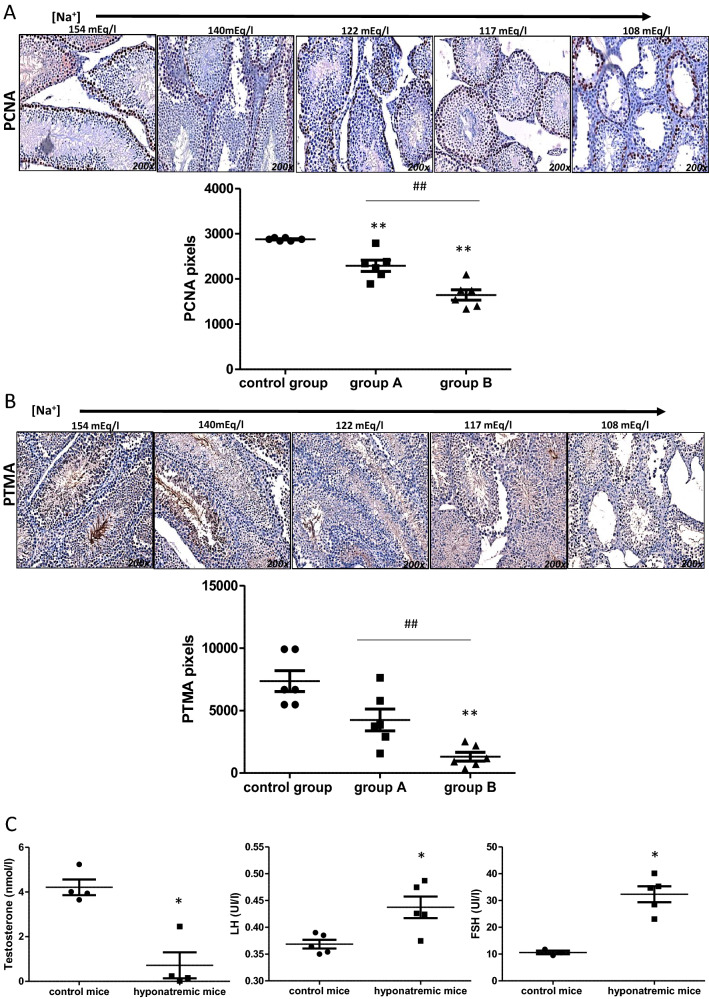
Immunohistochemical and hormonal assessment analysis of testes from control mice and hyponatremic mice. **A** Immunohistochemical analysis of PCNA. In the scatter plot, densitometric analysis of positive pixels of PCNA positive cells is represented (**p ≤ 0.02 group A and B vs. control group; ^##^*p* ≤ 0.02, group A vs. B). Results are expressed as mean ± SD. **B** Immunohistochemical analysis of PTMA. In the scatter plot, densitometric analysis of positive pixels of PTMA-positive cells is represented (**p ≤ 0.02 group A and B vs. control group; ^##^*p* ≤ 0.02, group A vs. B). Results are expressed as mean ± SD. **C** Hormonal evaluation of testosterone (nmol/L), LH (UI/L) and FSH (UI/L) from mice with [Na^+^] ≤ 140 mEq/L (hyponatremic mice) normalized vs. mice from control group. Results are expressed as mean ± SD (*p ≤ 0.05). For immunohistochemical images: [Na^+^] 154 mEq/L is from control group; [Na^+^] 140 and 122 mEq/L are from group A, T3 and T8, respectively [Na^+^] 117 and 108 mEq/L are from group B, T8 and T14, respectively

Testicular weight was reduced in hyponatremic mice (*p* ≤ 0.02 vs. control group). Histological examination indicated that the size of seminiferous tubules was reduced (*p* ≤ 0.05 group A and group B vs. control group and group A vs. group B*)*. This was a consequence of the reduction of the thickness of the epithelium (*p* ≤ 0.002 group B vs. control group and *p* ≤ 0.05 group A vs. group B), whereas the lumen area appeared increased (*p* ≤ 0.02 group B vs. control group and *p* ≤ 0.05 group A vs. group B) (Fig. 6a, c). Furthermore, the expression of the Proliferating Cell Nuclear Antigen (PCNA) gene (Fig. 7a), which is involved in DNA replication, and of the Prothymosin alpha gene (PTMA) (Fig. 7b), which is involved in the maturation of spermatogonia into primary spermatocytes, was reduced in animals with hyponatremia (*p* ≤ 0.02 vs. control group and group A vs. group B). Hormonal evaluation revealed that hyponatremic mice had lower serum testosterone levels than normonatremic mice and higher serum FSH and LH levels (*p* ≤ 0.05 vs. control group) (Fig. 7c).

## Discussion

Clinical data have shown that the effects of hyponatremia extend beyond neurological alterations and a rat model of hyponatremia has confirmed for instance that this condition is associated with a marked reduction of bone density [17]. Further analyses performed in this model indicated that other alterations occur in hyponatremic animals (e.g., hypogonadism, sarcopenia and cardiomyopathy).

We have developed a model of hyponatremia secondary to SIAD in male mice by administering a liquid diet, with the addition of continuous infusion of dDAVP via osmotic mini-pumps. Weight fluctuations were observed in animals during the two weeks in which dDAVP was administered at two different rates. Overall, the final weight was significantly increased at the end of this period both in mice that received 0.3 ng/h or 0.5 ng/h dDAVP. The final increase was around 10–15%, in agreement with what had been observed in other mouse or rat models of SIAD, in which similar infusion rates of dDAVP had been used [23, 28]. The final weight gain very likely indicates a condition of water retention, considering also that a significant weigh reduction was observed after the removal of dDAVP mini-pumps.

Accordingly, serum [Na^+^] progressively decreased in mice that received dDAVP, with lowest mean concentrations of 123.4 mEq/L and 111.7 mEq/L in animals receiving an infusion rate of 0.3 ng/h and 0.5 ng/h, respectively. Only in rats lower serum [Na^+^] had been obtained after two weeks of dDAVP infusion (106 mEq/L), yet using a much higher dDAVP infusion rate (5 ng/ml) [28]. Serum [Na^+^] reduction was paralleled by a decrease of serum osmolality. The discontinuation of dDAVP administration led to a rapid increase of both serum [Na^+^] and osmolality.

As per urine parameters, a marked volume reduction occurred soon after the insertion of dDAVP mini-pumps, followed by a recovery at the end of administration. Accordingly, a significant increase of urine osmolality was observed after the initiation of dDAVP infusion, with a peak after 3 days, followed by a progressive reduction, with a nadir after the discontinuation of the infusion. A similar early increase of urine osmolality, followed by a progressive decrease, had been reported previously in the rat model of SIAD [28]. With regard to sodium excretion, a marked increase was observed in the first 3 days of dDAVP infusion, followed by a subsequent reduction. No differences in urine parameters were observed in the control group. The aforementioned findings are in agreement with a condition of hyponatremia secondary to SIAD.

Upon sacrifice, tissues were excised at different time points in control animals and in animals receiving dDAVP. In the present study, findings regarding liver and testis alterations associated with hyponatremia have been reported.

We first focused on the liver, because signs of steatosis were already evident at an initial macroscopic examination. Noteworthy, no data regarding liver alterations in animal models of hyponatremia had been previously published. On clinical grounds, it is well known that hyponatremia is a negative prognostic factor in patients with cirrhosis [29, 30]. In tissue sections, lipid droplets’ deposition was present starting from a serum [Na^+^] of 140 mEq/L, corresponding to a condition of mild hyponatremia in this animal model. Interestingly, lipid accumulation progressively increased as serum [Na^+^] dropped down. Together with these morphological changes, we observed that the expression of SREBP-1 increased in hyponatremic mice. SREBP-1, together with SREBP-2, modulates lipogenesis. In particular, SREBP-1 induces the expression of the hydroxy-methyl-glutaryl-CoA synthase enzyme, which is the rate-limiting enzyme in the biosynthesis of cholesterol [31]. Accordingly, PPAR-α and PPAR-γ expression were increased in hyponatremic mice. PPARs are ligand inducible transcription factors under the class of nuclear receptors, which are involved in different physiological functions, including lipid metabolism. Different PPAR isoforms (i.e., α, γ, ẟ) have been associated to the pathogenesis of liver steatosis in humans [32]. Non-alcoholic fatty liver disease (NAFLD) represents the first step of a broad spectrum of alterations that lead to the more severe forms of non-alcoholic steatohepatitis (NASH), fibrosis, and ultimately cirrhosis, liver failure and hepatocarcinoma [33]. Interestingly, in hyponatremic mice liver fibrosis was also observed, as indicated by Sirius red staining. In addition, the expression of α-SMA and of CTGF protein, which are markers of myofibroblast formation, eventually leading to liver fibrosis, appeared increased [34, 35].

The etio-pathogenesis of liver steatosis is multifactorial and still incompletely understood. Among the factors that have been identified as effective triggers of steatosis, a genetic predisposition, environmental factors, metabolic alterations, inflammation and molecular mechanisms have been described [33]. One of the key molecular factors that have been related to liver steatosis is oxidative stress. Noteworthy, antioxidants, such as flavonoids, curcumin, vitamin E and resveratrol, have been proposed for the treatment of NAFLD [36]. Interestingly, we and others have previously observed oxidative stress induction in cells exposed to low [Na^+^] [18, 37–40]. Noteworthy, high levels of expression of HMOX 1, an inducible oxidative stress protein, were detected in the liver of hyponatremic mice. Further evaluation, by comparing serial liver sections and using cell specific markers, indicated that the cells expressing HMOX 1 were activated Kupffer cells and HSC. In the liver, Kupffer cells, which belong to the family of macrophages, are localized within the lumen of liver sinusoids. When activated, they produce inflammatory cytokines, TNF-alpha, oxygen radicals, and proteases, which may ultimately lead to liver injury [41]. HSC, also known as Ito cells, are quiescent peri-sinusoidal cells that, once activated, acquire proliferative and contractile activity, and contribute to extracellular matrix production. As such, activated HSC represent the major cell type involved in liver fibrosis [42].

Overall, these data suggest for the first time that hyponatremia may be another factor contributing to the onset of liver steatofibrosis. It is worth mentioning that sodium glucose cotransporter 2 (SGLT-2) inhibitors, a recent class of antidiabetic medications, have been shown to effectively attenuate inflammation, oxidative stress and fibro-genesis and clinical trials involving the use of these drugs in NAFLD are ongoing [43, 44]. Noticeably, SGLT-2 inhibitors have been found to correct serum [Na^+^] in patients with hyponatremia secondary to SIAD, by inducing osmotic diuresis [45, 46]. These unpredicted effects of SGLT-2 inhibitors appear of particular interest, also in view of the fact that liver failure is a very well-known etiology of hyponatremia due to fluid retention [1].

In this study, we also analyzed the testes of hyponatremic mice. Testicular alterations had been previously reported in a rat model of hyponatremia secondary to SIAD [20]. The authors found that the weight of testes in hyponatremic mice was significantly reduced compared to control mice. At histological examination, impaired spermatogenesis, tubular atrophy and fibrous degeneration were observed in the testes of animals with low serum [Na^+^]. From a hormonal point of view, after 10 weeks of hyponatremia, rats developed a moderate reduction of serum testosterone, with a significant increase of FSH and LH [20]. Hence, we were interested to verify whether similar alterations could be confirmed in our mouse model of hyponatremia. Interestingly, our data were completely in agreement with the data observed in rats. The weight of testes was significantly reduced in hyponatremic mice and tubular atrophy was observed. In particular, the thickness of the seminiferous epithelium was decreased, whereas the lumen area appeared increased. The expression of PCNA, which is a nuclear protein involved in DNA replication, elongation and repair [47], was reduced in the testes of mice with hyponatremia. PCNA also controls cell cycle progression through the G_1_/S boundary by direct interaction with cyclin/cdk [48]. In the testis, PCNA is mainly expressed in the nuclei of mitotic active spermatogonia and of primary spermatocytes [49]. Reduced expression of PCNA has been associated to germinal arrest [50]. Furthermore, in our model, hyponatremia was associated with a reduced expression of PTMA, which is a small peptide expressed in pre- and post-meiotic phases of spermatogenesis. Its presence in the heads of spermatids and epididymal spermatozoa, associated with the acrosome system, supports a role of PTMA also in mature germ cells function [51]. It is with mentioning that, although Leydig cell alterations have been reported in the testes of Foxn1^nu/nu^ mice, no germ cell abnormalities have been observed [52]**.** In addition, low-level testosterones have been found in Foxn1^nu/nu^ mice [53]. In our model, we detected in normonatremic mice similar testosterone levels to those previously reported in Foxn1^nu/nu^ mice [52]. However, lower levels of testosterone were observed in hyponatremic mice, together with higher levels of FSH and LH. These findings are in agreement with previous observations in a rat model of hyponatremia [20]. Admittedly, our data confirm that hyponatremia is associated with atrophy of the seminiferous epithelium and primary hypogonadism. Noteworthy, we described here for the first time that hyponatremia is associated with the onset of liver steatofibrosis, a potential precursor of liver cirrhosis and cancer [33]. These data further extend the variety of previously unpredicted extra-neurological alterations associated with hyponatremia. Of course, these observations need to be confirmed in clinical practice. If so, clinicians would have additional reasons to thoroughly evaluate patients with hyponatremia and to promptly correct this electrolyte alteration. Furthermore, additional analyses will be performed in upcoming studies addressing other tissues obtained from hyponatremic mice.

## Declarations

### Research involving animals

All animal experiments were conducted according to institutional ethical norms and national laws after approval from the Italian Ministry of Health [D. N° 688/2020-PR (prot. 17E9C.201)].
